# Interventions to increase young people's interest in STEM. A scoping review

**DOI:** 10.3389/fpsyg.2022.954996

**Published:** 2022-10-11

**Authors:** Milagros Sáinz, Sergi Fàbregues, María José Romano, Beatriz-Soledad López

**Affiliations:** ^1^Internet Interdisciplinary Institute, Universitat Oberta de Catalunya, Barcelona, Spain; ^2^Department of Psychology and Education, Universitat Oberta de Catalunya, Barcelona, Spain

**Keywords:** interest, interventions, gender, secondary education, stem

## Abstract

The underrepresentation of young people and particularly young women in many STEM fields has inspired various intervention programmes and research intended to boost their interest in these areas. The purpose of this scoping review is to examine the characteristics and effectiveness of interventions designed to encourage interest in STEM among secondary school students, particularly female students, over the past 20 years. A systematic search of the literature in five databases and additional search strategies resulted in identifying 215 studies evaluating interventions in different disciplinary fields. Data extraction and synthesis of these studies were carried out, focusing on the methodologies and theoretical foundations used. Twenty-five exemplars were selected to illustrate best practices in designing and evaluating interventions that address the various facets of young people's lack of interest in STEM. These interventions attempt to modify and/or manipulate multiple environmental and school factors to impact students' personal factors associated with STEM interest, such as achievement, self-perception of ability, and self-efficacy. Implications for the design of future interventions and potential outcomes are then discussed.

## Introduction

The under representation of women in some Science, Technology, Engineering, and Mathematics (STEM) disciplines, such as the physical sciences, computer science, and engineering, is common throughout Western countries (UNESCO, 2018; Sáinz, 2020). Despite numerous efforts to promote equal opportunities, several studies have confirmed the persistence of sexist beliefs regarding the competences that men and women must possess to access and develop certain academic and professional activities (Leaper and Brown, 2014; Sáinz et al., 2020). These beliefs, which discourage young women from pursuing non-traditional STEM career pathways, revolve around the idea that women do not have sufficient technological and mathematical capabilities (Wang and Degol, 2013; Sáinz et al., 2020).

Many initiatives and interventions (i.e., empirical investigations that manipulate an independent variable and follow the effect over time) have been conducted worldwide to engage young women in STEM, especially in those STEM disciplines with a higher under-representation of women (Liben and Coyle, 2014; Rosenzweig and Wigfield, 2016; van den Hurk et al., 2019; Prieto-Rodriguez et al., 2020). Research has also highlighted the importance of analyzing STEM disengagement in order to improve the design of interventions, thereby attracting and retaining women in STEM pathways (Rosenzweig and Wigfield, 2016).

Interventions tend to represent operationalized theory in action. For some authors, they characterize the testing of a theory as applied in a given educational context (Lazowski and Hulleman, 2016). From a practical point of view, intervention-based studies expand our understanding of which intervention components are most effective in raising students' interest in STEM and how this can be sustained in the long term (Lazowski and Hulleman, 2016; Rosenzweig and Wigfield, 2016). Such understanding can guide educational policies and provide recommendations informed by scientific evidence (Liben and Coyle, 2014; Lazowski and Hulleman, 2016; Rosenzweig and Wigfield, 2016). For this reason, this review builds on previous systematic reviews of STEM intervention studies. These previous reviews have emphasized the importance of drawing on clear theoretical frameworks when designing and implementing a proper evaluation of an intervention's effectiveness, and how the various intervention features and components take effect (Rosenzweig and Wigfield, 2016; van den Hurk et al., 2019; Prieto-Rodriguez et al., 2020).

There is no single factor that alone can influence on girls' and women's participation, achievement, and progression in STEM education (Wang and Degol, 2013; UNESCO, 2018). For this reason, van den Hurk et al. (2019) in their review have categorized STEM intervention studies according to the factors they address: environmental level (such as stereotypical cultural and societal beliefs about gender and STEM, or the lack of female role models in STEM); school level (such as educational policies, school climate, teachers' beliefs and attitudes, or pedagogy); and student level (including cognitive characteristics such as academic ability and achievement, background characteristics such as gender and socioeconomic status, or affective characteristics, such as self-efficacy, motivation, belonging, and engagement). All these factors not only correlate with interest and persistence in STEM education, but they are also interrelated (Blickenstaff, 2005).

Personal level factors involved in shaping young people's (particularly girls') engagement and interest in STEM have been prioritized and measured by many intervention studies analyzed in prior reviews (Rosenzweig and Wigfield, 2016; van den Hurk et al., 2019; Prieto-Rodriguez et al., 2020). In this regard, Rosenzweig and Wigfield (2016) review of 53 intervention studies published between 1985 and 2015 focused on the following five motivation-related categories: competence-related beliefs (such as self-concept of ability, self-efficacy, confidence, and outcome expectations); beliefs about value, interest, and intrinsic motivation; attributions about academic success and failure; beliefs about intelligence; and achievement goal orientation. Many intervention studies address personal factors related to students' performance and engagement in STEM through changes either in school level factors, social-environmental level factors, or a combination of them. Interestingly, Prieto-Rodriguez et al. (2020) systematic review concluded that successful activities encouraging girls' STEM identity formation combined both inclusive curriculum and pedagogies (strategies at the school level) and exposure to female role models (strategy at the environment level).

Current societal stereotypes about the type of students who are expected to succeed in STEM (e.g., middle-class white male students) discourage many students who do not meet these attributes (e.g., girls, students from low SES or migrant families, as well as non-white students or students with disabilities) from entering in STEM fields (Good et al., 2003; Rosenzweig and Wigfield, 2016; Sáinz and Müller, 2018). However, in Western societies and contrary to students from low-SES families or with migrant and ethnical backgrounds, girls are not a minority in the school context (UNESCO, 2021). Moreover, females in most Western countries are highly represented in STEM disciplines that align with the caring role associated with feminine roles, such as medicine, chemistry, or biology (UNESCO, 2018).

In addition, several meta-reviews and reviews (i.e., Wang and Degol, 2013; van den Hurk et al., 2019) suggest the influence of school level factors in the teaching and learning of STEM subjects on girls' engagement, achievement, and progression in STEM. The instructional approach of STEM teachers, the curriculum of STEM subjects, or teachers' beliefs, attitudes, behaviors, and interactions with students can positively influence girls' performance and engagement with STEM education and their interest in pursuing STEM careers (UNESCO, 2018). Similarly, female students are frequently attributed less competence by their male peers in STEM activities developed in the classroom (Sáinz, 2020).

While some of these prior reviews have laid the theoretical groundwork for the design of present and future intervention studies (Rosenzweig and Wigfield, 2016; van den Hurk et al., 2019), they have failed to provide a comprehensive account of the alignment between the different methods and theories used to raise young people's interest in STEM. Moreover, previous reviews lack an in-depth analysis of the range of methods and methodological approaches used to evaluate the effectiveness of interventions. In the present review, we attempt to fill this literature gaps by focusing on the methods and theories used in intervention studies to increase young people's interest in STEM. In addition, this review will focus on intervention studies aimed at increasing girls' STEM motivation, while it also considers the intersection of gender with other inequality variables (such as race/ethnicity, and SES level). The findings of this review will shed light on how to enhance the design and implementation of interventions aimed at closing the gender gap in STEM pathways.

### Challenges to the evaluation of the interventions

Several systematic reviews of interventions have highlighted a need for research into the effectiveness of intervention programmes intended to attract and retain highly motivated students in STEM fields (van den Hurk et al., 2019; Kolne and Lindsay, 2020; Prieto-Rodriguez et al., 2020). However, van den Hurk's (2019) systematic review of empirical studies on the effectiveness of STEM-related interventions published between 2005 and 2017, raised an important issue: only a few of these evaluations were adequately designed to determine whether the observed effects were actually caused by the intervention (van den Hurk et al., 2019). In many of the instances, the studies under review were neither randomly selected nor applied a control group.

Liben and Coyle's (2014) review of gender developmental interventions addressing the STEM gender gap provided a taxonomy of five intervention goals (remediate, revise, refocus, re-categorize, and resist) designed to enhance the alignment between (a) cognitive, personal, and/or perceived qualities of girls and women and (b) the demands and opportunities of STEM. In agreement with this review, many of the identified interventions were not systematically evaluated and therefore provided little empirical evidence of whether they successfully engaged girls and women in STEM-related subjects, especially in the long term. In this sense, it has been acknowledged that long-term interventions can contribute not only to raising, but also to maintaining young people's interest in STEM (Liben and Coyle, 2014; van den Hurk et al., 2019). According to Harackiewicz and Priniski (2018), the primary outcomes targeted by an intervention may not only serve as a measure of efficacy but can also trigger positive recursive processes that drive longer-term impacts. In fact, all the systematic reviews in the literature concluded that long-term interventions or repeated participation in interventions were most likely to result in meaningful engagement in STEM (Liben and Coyle, 2014; Rosenzweig and Wigfield, 2016; van den Hurk et al., 2019; Prieto-Rodriguez et al., 2020).

In their review of psychosocial-based interventions in higher STEM education, Harackiewicz and Priniski (2018) identified the following psychological processes as critical for various educational outcomes in higher education: students' lack of interest in certain STEM topics and subjects; students' lack of confidence in their own abilities; students experiencing identity threat in certain fields; students doubting about the suitability of an academic discipline, or about the fact that they belong to a particular STEM career pathway; students experiencing a cultural mismatch between institutional norms and their own values; and students suffering from various emotional issues.

In addition, Rosenzweig and Wigfield (2016) review on STEM motivation interventions also discussed about the need of understanding the impact of individual and contextual factors (moderators) to better disentangle their influence on the effects of interventions. In a similar fashion, Kolne and Lindsay (2020) systematic review analyzed the impact of programmes and interventions in increasing interest and participation in STEM education and careers among children and young people with disabilities. These authors concluded that more controlled designs are needed to determine the impact of specific intervention components and participant characteristics, such as gender and students' disabilities, on the evaluation of the intervention effectiveness.

### The present review

The purpose of this scoping review is to examine the characteristics and content of intervention studies aimed at increasing young people's participation in STEM (female students in particular), conducted in various geographical areas over the past 20 years. The present review builds on prior systematic reviews of STEM interventions that have identified strategies for change that emerge from outstanding theories (Liben and Coyle, 2014; Rosenzweig and Wigfield, 2016). Additionally, it expands on prior reviews that have identified a set of factors (at the social-environmental level, at the school level, and at the student level) steering students' decisions to pursue STEM education or not (van den Hurk et al., 2019).

While most reviews to date have evaluated the effect of interventions on increasing interest in STEM, only a few have explored of the way the methodological and theoretical approaches have been applied and combined (i.e., Rosenzweig and Wigfield, 2016; Prieto-Rodriguez et al., 2020). We attempt to bridge this research gap by bringing together a scoping review of publications evaluating interventions or initiatives to increase young people's STEM participation, with particular attention on those that target girls. In addition, in alignment with previous reviews (Rosenzweig and Wigfield, 2016; van den Hurk et al., 2019), we will identify through a selection of interventions what type of school and social-environment level strategies have been used in order to tackle various personal factors influencing young people's interest in STEM pathways. Providing policymakers and educational practitioners with better information about the characteristics of effective STEM initiatives may help increase engagement and participation in STEM. It will also enable policymakers and practitioners to select the type of initiative that best suits their particular needs and interests (Australian Education Council, 2019).

Consistent with previous research (Rosenzweig and Wigfield, 2016; van den Hurk et al., 2019; Prieto-Rodriguez et al., 2020), this review focuses on middle and high school, the educational stages where decisions about academic and career pathways take place. Therefore, the primary goal of this review is to examine the main features and strategies deployed by a selection of intervention studies in order to inform the design of future initiatives to increase girls' interest in STEM. The questions this review will address are as follows:

- **R.Q.1**. What are the characteristics of research (interventions) aimed at increasing interest in STEM subjects and/or careers and reduce the underrepresentation of girls in STEM?
- **R.Q.2**. What methods have been used to measure the effectiveness of these interventions?
- **R.Q.3**. Which intervention studies are the best examples for inspiring and guiding the design of future interventions?

A systematic search of the literature in five databases and additional search strategies were used to respond to the three afore-mentioned research questions. More specifically, the ultimate goal of this review is to examine existing methodological and theoretical gaps in the different identified interventions. This could provide guidelines and recommendations for the design and development of future interdisciplinary intervention strategies.

## Materials and methods

### Design

A scoping review methodology was used (Arksey and O'Malley, 2005) in this study. This approach is particularly useful when, as in this review, researchers are interested in identifying the scope and extent of published research on a particular research topic and in examining how this research has been carried out (Arksey and O'Malley, 2005; Grant and Booth, 2009; Munn et al., 2018). Since the purpose of our review was to identify knowledge gaps and scope a body of literature about STEM intervention studies to raise preferably girls' interest in STEM—rather than producing a synthesized answer to a particular question— we chose to carry out a scoping review instead of a systematic review (Munn et al., 2018). Thus, the scoping review was the most suitable systematic reviewing methodology to determine the coverage of the wide range of literature that evaluates STEM interventions for secondary students, to provide a detailed overview of this literature, and to identify the most important literature gaps. In the conduction and reporting of this review, we adhered to the Preferred Reporting Items for Systematic Reviews and Meta-Analyses guidelines for scoping reviews (PRISMAScR). These guidelines have been outlined by Tricco et al. (2018). These guidelines, outlined by Tricco et al. (2018), include, among others: specifying the characteristics of the sources of evidence used as eligibility criteria and providing a rationale; describing all information sources in the search as well as the date of the most recent search; presenting the complete electronic search strategy for at least one database, including any limits used; and describing the process for selecting sources of evidence included in the scoping review. When preparing the methods section and the remaining sections of the review, we ensured that all of the aforementioned guidelines of the PRISMA-ScR were followed.

### Search strategy

A systematic search of empirical literature published in English between 1998 and 2019 was carried out in the following five databases: APA PsycNET, ERIC, ProQuest, Scopus, and Web of Science. These databases were selected because of their broad coverage of literature on science and technology, education, behavioral sciences and mental health, social sciences, the arts, and the humanities. The searches were carried out in the title and abstract fields using search terms associated with the following four concepts: interventions, STEM studies and professions, outcomes, and gender. The search query used, developed with the assistance of an information scientist from the Universitat Oberta de Catalunya, is shown in Table 1.

**Table 1:** Search.

**Concept**	**Search terms (in title or abstract)**
Intervention	Program* OR Interven* OR Initiative* OR Strateg* OR Seminar* OR Workshop* OR Course* OR Session*
STEM studies and professions	STEM OR Math* OR Science* OR Scient* OR Engineer* OR Technolog* OR “Technical stud*” OR career* OR “Technical career*” OR “Technical occupation*” OR “Technical subject*” OR “Scientific career*” OR “Scientific stud*” OR “Scientific occupation*” OR “Scientific subject*”
Positive outcomes	Interest* OR Engag* OR Motivat* OR Perform* OR Score* OR Grade* OR Abilit* OR Achiev* OR Choice* OR Selection OR Self-efficacy OR “Self-competence*” OR “Self-perception* of abilit*” OR “Sense of belonging” OR Stereotyp* OR Attitude* OR Participat* OR Involv* OR Capab* OR Encourag* OR Increas* OR Aspiration*OR “Self-concept*”
Gender	Gender OR Girl* OR Female* OR Woman OR Women OR Sex

Three additional search strategies were used to complement the database search. First, 20 influential journals in the social and behavioral sciences were hand searched (see Table 2): Second, lists of publications from influential authors in the field were reviewed for studies not identified in the database search. Third, citation searching was carried out by scanning the references cited by key articles.

**Table 2:** Influential journals in the social and behavioral sciences.

**Journal name**	**Impact factor (Journal citation reports, 2021)**
American Psychologist	16.358, Q1, Psychology, Multidisciplinary, 4/147
Annual Review of Psychology	27.782, Q1, Psychology, 1/79
Developmental Psychology	4.497, Q2, Psychology, Developmental, 19/78
Educational Psychology Review	8.240, Q1, Psychology, Educational, 1/61
Educational Research	2.968, Q2, Education & Educational Research, 90/267
International Journal of Science Education	2.518, Education & Educational Research, 127/267
Journal of Applied Developmental Psychology	3.280, Q2, Psychology, Developmental, 33/78
Journal of Educational Psychology	6.856, Q1, Psychology, Educational, 4/61
Journal of Experimental Child Psychology	2.547, Q3, Psychology, Developmental, 47/78
Journal of Personality and Social Psychology	8.460, Q1, Psychology, Social, 3/65
Personality and Social Psychology Bulletin	4.560, Q2, Psychology, Social, 18/65
Perspectives on Psychological Science	11.621, Q1, Psychology, Multidisciplinary, 6/147
Psychological Bulletin	23.027, Q1, Psychology, 3/79
Psychological Science	10.172, Q1, Psychology, Multidisciplinary, 9/147
Psychology of Women Quarterly	4.292, Q1, Psychology, Multidisciplinary, 33/147
Review of Educational Research	13.551, Q1, Education & Educational Research, 1/267
Science	63.798, Q1, Multidisciplinary Sciences, 2/73
Sex Roles	3.812, Q2, Psychology, Developmental, 25/78
Social Psychological and Personality Science	5.316, Q1, Psychology, Social, 12/65
Social Science Quarterly	1.781, Q3, Political Science, 106/187

### Inclusion and exclusion criteria

In order to be included in the review, publications had to meet four inclusion criteria. First, they needed to report empirical research evaluating interventions for promoting the participation of secondary school students in STEM fields. Second, they needed to describe the aims, participants, and context of the intervention and provide a succinct description of its implementation. Third, they needed to evaluate the effectiveness of the intervention through clearly defined and operationalized outcomes using either quantitative, qualitative, or mixed methods. Fourth, they needed to be in English and published between 1998 and 2019. All types of publications were included, including journal articles, books, book chapters, and dissertations. Studies in which the participants were the parents or secondary school teachers of students were also included. Non-empirical articles, such as systematic reviews, editorials, or commentaries, were excluded.

### Study selection

The publications retrieved from the databases and those identified through the complementary search strategies were imported into the EPPI Reviewer software, which was used to facilitate the study selection. The selection was carried out in two phases. In Phase 1, two researchers independently screened a random sample of 10% (*n* = 4.017) of the articles. Each reviewer screened the same number of articles and disagreements between the two researchers were resolved through discussion with the involvement of a third reviewer, when necessary. Inter-rater agreement was high (Kappa = 0.825). The remaining articles were divided between the two researchers. In Phase 2, the full text of the eligible publications was independently reviewed by the same two researchers. Disagreements in this phase were again resolved by consensus. The percentage of discrepancies between the two researchers during the screening and eligibility phases was similar.

### Data extraction and synthesis

The steps described by Arksey and O'Malley (2005) were followed during the data extraction and synthesis. First, data from the included publications was extracted in Excel using a standardized tool. The following information was collected: publication metadata (i.e., publication year and type affiliation), intervention characteristics (i.e., purpose, participants' profile, setting, and theory motivating the intervention), focus of the evaluation, and methodological features of the study. Two researchers independently performed the data extraction. Disagreements between the researchers were discussed among the members of the research team until a consensus was reached. Second, once the extraction was completed, summary tables were generated to chart the extracted data and compare between intervention study types. This comparison allowed the researchers to identify patterns across the included studies and generate a narrative account of the results. At this point, researchers returned to the original publications several times to ensure that the summaries were supported by the data.

## Results

**Figure 1:**
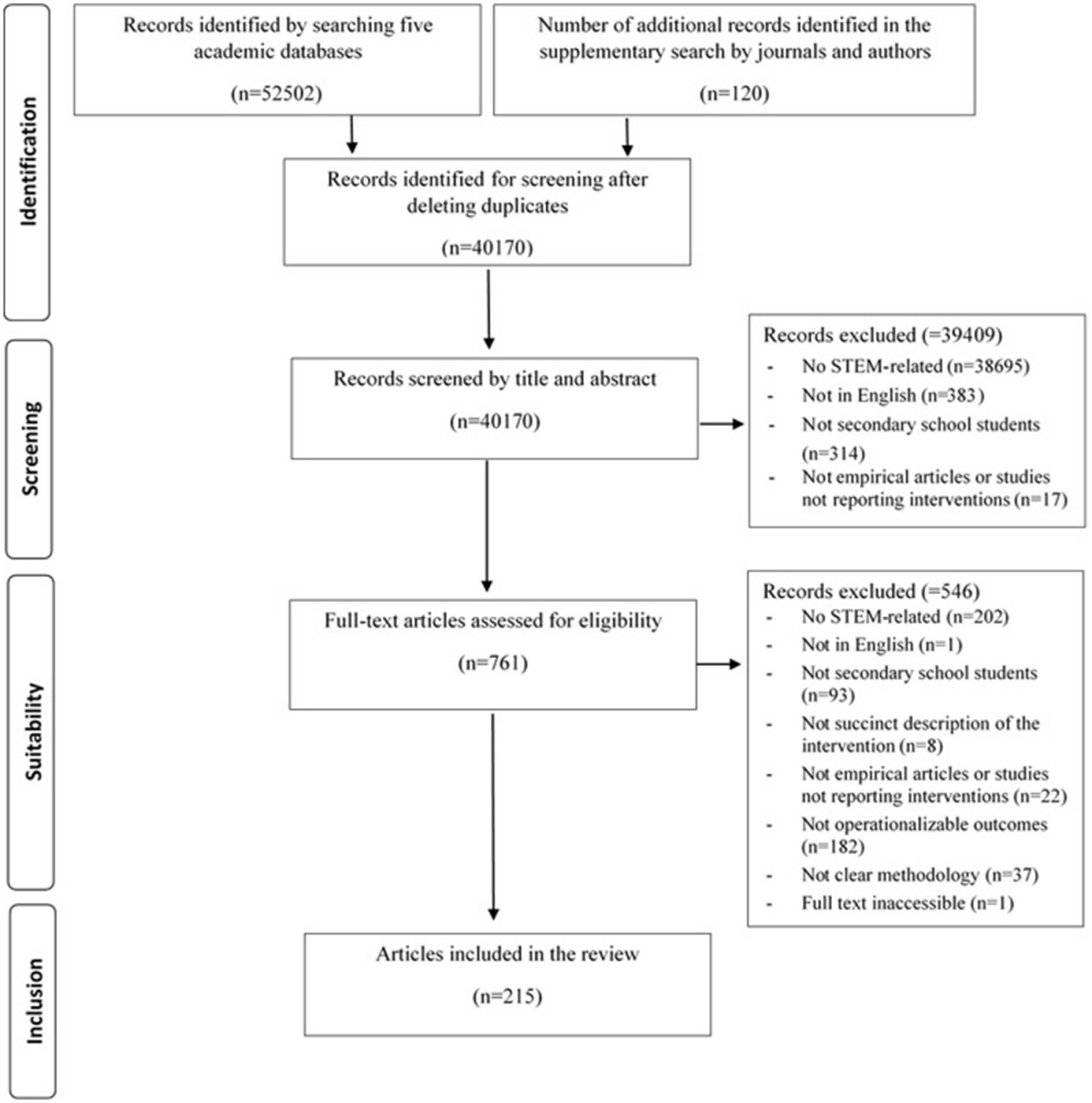
Flow-chart diagram illustrating inclusion and exclusion of articles during search process.

The database search generated 52.622 publications, of which 52.502 were identified through database searching, while 120 were identified using the additional search strategies. After removing duplicate publications and assessing eligibility, 215 publications were included. Figure 1 shows the Preferred Reporting Items for Systematic Reviews and a Meta-Analyses (PRISMA) flow diagram of the review process.

### R.Q.1. Characteristics of the publications and the interventions

Most of the publications (*n* = 180) were published between 2010 and 2019 (see Table 3). The majority of publications were journal articles (*n* = 147), followed by conference proceedings (*n* = 34), and dissertations (*n* = 25).

**Table 3:** Characteristics of the publications.

	***n***	**%**
**Year of publication**
2000–2009	35	16.3
2010–2019	180	83.7
**Type of publication**
Journal article	147	68.4
Dissertation	25	11.6
Book chapter	7	3.3
Conference proceedings	34	15.8
Working paper or report	2	0.9
**Corresponding author discipline**
Arts & humanities	6	2.8
Education	47	21.9
Psychology	63	29.3
Science (STEM)	45	20.9
Technology (STEM)	48	22.3
Social sciences	6	2.8

Categories are mutually exclusive. The percentages are calculated relative to the number of articles that included information on this feature.

The main disciplines of corresponding authors were psychology (*n* = 63), STEM fields with a high technological component (i.e., engineering, computer science, and information technologies, *n* = 48), education (*n* = 47), and STEM fields with a high scientific component (*n* = 45) including mathematics, the physical sciences, medicine, pediatrics, chemistry, biomedicine, agriculture, and biology.

As shown in Table 4, the majority of intervention studies (*n* = 95) focused on scientific STEM fields, mainly biology, the physical sciences, mathematics, and chemistry, while others *n* = 45) considered STEM fields with a high technological component (i.e., computer science or engineering). Many of the interventions (*n* = 71) did not identify any STEM field. Only three included a combination of arts and STEM competences (STEAM).

**Table 4:** Characteristics of the interventions.

	***n***	**%**
**STEM field**
Science	95	44.2
Technology	45	20.9
Various STEM disciplines	71	33.0
STEAM	4	1.9
**Geographical location**
Africa	3	1.4
Asia	14	6.5
Europe	43	20.0
Latin America	1	0.5
Latin America & Africa	1	0.5
North America	149	69.3
Oceania	4	1.9
**Target people**
Only students	176	81.9
Students & parents	8	3.7
Students & teachers	21	9.8
Students & mentors (peers or professionals)	5	2.3
Combination of several previous categories	5	2.3
**Gender**
Boys and girls	146	67.9
Only girls	66	30.7
Combination of the previous	3	1.4
**Duration**
Short-Term	85	39.5
Mid-Term	72	33.5
Long-Term	58	27.0
**Type of activity**
Ordinary classroom activity	119	55.3
Extracurricular activity	91	42.3
Both	5	2.3
**Ordinary classroom activity**
Hands-On classroom activities	100	84.0
Workshops	5	4.2
Laboratory experiments	4	3.4
Excursions	4	3.4
Games	4	3.4
Counseling sessions	2	1.7
**Extracurricular activities**
Summer camps	33	36.3
Competitions	2	2.2
Out of school/after school activities	24	26.4
University camps	10	11.0
Workshops, Presentations	22	24.2
**Theory feeding the intervention**
Yes	127	59.1
No	88	40.9

In terms of geographical location, the majority (*n* = 149) of the studies were conducted in North America, particularly the United States. Likewise, most of the interventions (*n* = 176) were conducted exclusively with students.

In addition, while most of the intervention studies (*n* = 146) targeted both genders, 66 focused solely on female students. Furthermore, 85 of the intervention studies had a short duration (1 or 2 h session, 1 day, or 1 week), and 72 had a mid-term duration (between 2 weeks and 4 months). Interestingly, 58 of the intervention studies were long term, lasting more than 14 weeks.

The vast majority of interventions were organized in the context of regular classroom activities (*n* = 119) and were hands-on (*n* = 100). Moreover, the greatest part of the extracurricular activities consisted of summer camps (*n* = 33), workshops and presentations (*n* = 22), and afterschool activities (*n* = 20). Finally, while the majority of intervention studies explicitly drew on theoretical foundations (*n* = 127), 88 did not declare any particular theoretical approach.

### R.Q.2. Methodology of the interventions

A great number of the intervention studies (see Table 5) were exclusively based on a quantitative methodology (*n* = 141), whereas the rest on a mixed methods approach (*n* = 73). Only one study had a qualitative nature, which relied on interviews for collecting and analyzing data. Most of the 141 quantitative studies, had a quasi-experimental design (73), and 39 had an experimental design. Interestingly, a high proportion of the quasi-experimental studies (*n* = 47) had a pre-experimental design. Among the experimental studies, most of them applied a single factor inter-subject design (*n* = 27). The methods employed included self-reported surveys (*n* = 77), achievement tests (*n* = 8), and grades (*n* = 1), or combined various quantitative methods (*n* = 55).

**Table 5:** Methodology of the intervention studies with a quantitative approach.

	***n***	**%**
**Method**		
Quantitative	141	65.6
Qualitative	1	0.5
Mixed methods	73	34.0
**Type of design in the quantitative studies**		
Experimental	46	32.6
Quasi-Experimental	67	47.5
Cohort	22	15.6
Cross-Sectional	4	2.8
Various designs	2	1.4
**Type of experimental design**		
Single factor inter-subject design: 2 groups with pre- & post-test or only post	27	58.7
Single factor inter-subject design: multi-group with pre- & post-test or only post-test	18	39.1
Single factor within-subject design: single group with pre- & post-test	1	2.2
**Type of quasi-experimental**		
Pre-Experimental design: single group with pre- &post-test	47	70.1
Quasi-Experimental design, with non-equivalent control group with pre-post-measurements	14	20.9
Quasi-Experimental design: interrupted time series, simple (1 group, pre- & post-test)	4	6.0
Quasi-Experimental design: interrupted time series, with non-equivalent control group (pre & post-test)	2	3.0
**Type of method used in the quantitative studies**		
Achievement tests	8	5.7
Self-Reported surveys	77	54.6
Grades	1	0.7
Various methods	55	39.0

Categories are not mutually exclusive. The percentages are calculated relative to the number of articles that included information on this feature.

Curiously (see Table 6), the majority of intervention designs that used a mixed methods approach were convergent designs (*n* = 63). Designs used in the quantitative strand of mixed methods studies were mostly quasi-experimental (*n* = 57). The majority of the quasi-experimental studies applied a pre-experimental design (*n* = 43). Among the experimental studies (*n* = 9), most of them (*n* = 7) applied a single factor inter-subject design. The designs used in the qualitative strand of mixed methods studies were largely qualitative description (*n* = 45).

**Table 6:** Methodology of the intervention studies with a mixed-method approach.

	***n***	**%**
**Type of design of the mixed methods studies**		
Convergent	63	86.3
Explanatory sequential	10	13.7
**Type of design of the quantitative strand of the mixed methods studies**		
Experimental	9	12.3
Quasi-Experimental	56	76.7
Cohort	7	9.6
Various designs	1	1.4
**Type of experimental design of the mixed methods studies**		
Single factor inter-subject design: 2 groups with pre- & post-test or only post	7	77.8
Single factor inter-subject design: multi-group with pre- & post-test or only post	2	22.2
**Type of quasi-experimental design of the mixed methods studies**		
Pre-Experimental design: single group with pre-post test	43	76.8
Quasi-Experimental design, with non-equivalent control group with pre-post-test	8	14.3
Quasi-Experimental design: interrupted time series, simple (1 group, pre- & post-test)	3	5.4
Quasi-experimental design: interrupted time series, with non-equivalent control group (Pre- & Post-test)	2	3.6
**Type of design qualitative strand of the mixed methods studies**		
Case study	1	1.4
Content analysis	5	6.8
Ethnography	1	1.4
Grounded theory	1	1.4
Phenomenology	1	1.4
Qualitative description	45	61.6
Non-reported design	19	26.0
**Type of method used in the quantitative strand of the mixed methods studies**		
Achievement tests	5	6.8
Self-Reported surveys	43	58.9
Various methods	25	34.2
**Type of method used in the qualitative strand of the mixed methods studies**		
Open-Ended questions	15	20.5
Interviews	16	21.9
Focus groups	5	6.8
Journal entries	2	2.7
Observations	1	1.4
Various qualitative methods	34	46.6

Categories are not mutually exclusive. The percentages are calculated relative to the number of articles that included information on this feature.

Methods used in the quantitative strand of the mixed methods studies were mostly based on self-reported surveys (*n* = 43), whereas the qualitative strand in the mixed methods studies were mainly based on interviews (*n* = 16), open-ended questions (*n* = 15), or on various methods used simultaneously (*n* = 34).

As shown in Table 7, 30 of these interventions drew on Expectancy-Value theories (i.e., Victor, 2005; Eccles, 2009), 22 on Constructivist Learning Theories, and 17 on Social Learning Theory (Bandura's Self-Efficacy theory). Only three were inspired by feminist theories and feminist pedagogy, critical mass, and intersectional theories. Interestingly, 27 of the intervention studies relied on more than one motivation theory (competence-related beliefs, expectancy-value, attributions, or theories of intelligence).

**Table 7:** Theories and outcomes associated with the interventions.

	***n***	**%**
**Theories feeding the interventions**
Expectancy-Value theory	30	14.0
Social learning theory (self-efficacy)	17	7.9
Social role theory	2	0.9
Role model theory	5	2.3
Sociocultural learning theory (Vygotsky)	4	1.9
Feminist theories	3	1.4
Self-Determination theory	2	0.9
Constructivist learning theories	22	10.2
Career development (Holland) theory	1	0.5
Theories of intelligence	7	3.3
Identity theories	3	1.4
Self-Affirmation theory	1	0.5
Theories of emotion	1	0.5
Attribution theory	1	0.5
Stereotype threat theory	1	0.5
Various theories	27	12.6
**Outcomes**
Achievement	14	6.5
Motivation	74	34.4
Gender stereotypes	2	0.9
Achievement & motivation	74	34.4
Achievement & emotion	2	0.9
Achievement, motivation, & identity	3	1.4
Motivation & identity	9	4.2
Motivation & gender stereotypes	19	8.8
Motivation & emotion	5	2.3
Achievement, motivation, gender stereotypes, & emotion	4	1.9
Achievement, motivation, gender stereotypes, identity, & emotion	1	0.5
Achievement, motivation, & gender stereotypes	4	1.9
Motivation, gender stereotypes, & identity	3	1.4
Achievement, motivation, & emotion	3	1.4
Achievement, motivation, gender stereotypes, & identity	1	0.5

Categories are mutually exclusive. The percentages are calculated relative to the number of articles that included information on this feature.

Curiously, some of the intervention studies that did not draw on any particular theory included learning and the acquisition of knowledge on specific STEM content. For instance, the study by Tarng et al. (2011) provided the basic concepts of synchrotron light sources, while other interventions focused on students' attitudes and interest in STEM subjects and careers (Christensen et al., 2014, 2015; Christensen and Knezek, 2017; Acuña et al., 2018) or STEM performance (Brown and Brown, 2019; Jordaan and Tavenga, 2019; Mostoli et al., 2019).

In line with the theoretical foundations of interventions, 74 measured various motivational constructs as outcomes to evaluate effectiveness: self-concept of ability, self-efficacy, perceived utility value of STEM subjects, interest in pursuing STEM studies, intrinsic value of STEM, and attainment. Only 14 intervention studies exclusively focused on achievement, whereas 75 interventions combined the use of different motivational and achievement-related constructs.

### R.Q.3. Classification of outstanding interventions

A total of 25 intervention studies were selected according to various attributes of the intervention (context of the intervention, gender of target people, and main findings), and the intervention evaluation (main purpose, theory feeding the interventions, and evaluation method). That is, the interventions met the following criteria (see Table 7).

- Interventions drawing on one or more theoretical approach since this information serves as an indicator of the operationalization of theory in practice (Lazowski and Hulleman, 2016; Rosenzweig and Wigfield, 2016).
- The methodological approach was based on a quantitative or mixed methods design, since it provides further insights about the tools used to conduct the evaluation of the intervention (Kolne and Lindsay, 2020; Prieto-Rodriguez et al., 2020).
- The design of the intervention included an experimental or a quasi-experimental design, as it informs about the quality of the evaluation of the intervention (Rosenzweig and Wigfield, 2016; van den Hurk et al., 2019; Prieto-Rodriguez et al., 2020).
- The duration of the intervention lasted either more than 2 weeks (a mid-term) or several months or years (long-term), since this informs about the sustainability and long-term strategy of the intervention (Prieto-Rodriguez et al., 2020).
- The purpose and/or research questions included a gender perspective, since they provide a clear commitment of the intervention to tackle the dearth of women in STEM pathways (Tannenbaum et al., 2019).

The majority of the selected exemplars applied to the field of science (8/25), computer science (6/25), math (5/25), and STEM (4/25). Only one intervention applied to the field of engineering and another one to physical science.

Most of the selected interventions attempted to improve girls' and students' STEM motivation, performance, skills or competences, and self-perceptions (self-efficacy). To achieve the purpose nine of the selected interventions applied a STEM training strategy through courses, workshops, and summer camps (9/25). Brock's quasi-experimental study developed in the context of a math workshop (Brock, 2017) helped to reduce the gender gap in math achievement between advanced students. Similarly, Isiksal and Askar's (2005) experimental study made use of two software programs and observed no significant gender differences in mathematics achievement and self-efficacy. Denner's (2007) quasi-experimental study based on a STEM training program focused on improving girls' computer skills revealed that girls improved their computer skills, knowledge about computers, and perceived social support. Girls' perception that boys do better than girls with computers was reduced. Likewise, in Hall-Lay's (2018) quasi-experimental study developed in the framework of STEM programs, students who participated in robotics programs scored significantly higher than students enrolled in other STEM-related programs. However, no gender differences were observed.

Paslov (2006) quasi-experimental study demonstrated that girls who participated in the program improved their self-efficacy and achievement in mathematics. Similarly, Scott et al. (2017) quasi-experimental study with a computer science preparatory course showed that girls from ethnic group's interest in computer science increased over time, despite their initial lack of interest. Male students showed higher interest and aspiration in computer science. There were no gender differences in course completion, but taking a course did not improve female students' likelihood of majoring in computer science. In Todd and Zvoch (2019a) experimental study, girls who participated in the summer camp scored higher in science efficacy and attitudes toward science than girls in the control group. No significant differences were observed in science interest and science identity. In a similar vein, Todd and Zvoch (2019b) quasi-experimental research showed that girls from affluent families participating in a summer science program increased their affinities in science over time. However, among girls from low income families their early gains in affinities in science diminished over time. Finally, Ziegler and Heller (2000) experimental study demonstrated how an attribution program in physics improved girls' performance as well as their motivation and self-related cognitions in physics.

Other two of the selected interventions included evaluations of single-sex and coeducational contexts (2/25). In this way, Drobnis (2010) quasi-experimental study consisting of a computer science summer course concluded that boys in the mixed gender group and girls in the only girls group had higher computer self-efficacy and higher gains in computer science scores than girls in the mixed gender group. Interestingly, in Schilling and Pinnell (2019) experimental study by allowing participants to explore engineering in a positive environment and encouraging them to work through challenges, participants built confidence in engineering.

Five of the selected interventions developed changes in the curriculum and pedagogy (5/25). In Cantley et al. (2017) quasi-experimental research the pedagogical mathematics tool used increased girls' interest and enjoyment of mathematics, but it had no significant change in boys' attitudes. Additionally, no significant gender differences in pre-intervention enjoyment scores were observed. Chiu's (2011) experimental study focused on the recognition of women in science and men in humanities, awareness of academic gender stereotypes, and development of unique selves when learning science. In comparison to the control group, students in the experimental group did not experience any change in their attitudes toward learning science. Boys in both the experimental and control groups increased the value attached to learning science throughout the pre-test phase. However, in the experimental group girls' value exclusively increased during the post-test phase.

Mayberry's (2015) quasi-experimental research found that female-oriented curriculum improved girls' interest and confidence in STEM careers. Similarly, McHugh et al. (2018) quasi-experimental study examined the influence of incorporating mathematical skills into the curriculum as a complement to science content on students' achievement and attitudes toward science. Females outperformed males in science, but there were no significant differences in achievement between students from high and low-needs schools. Remarkably, Werner's (2017) quasi-experimental study deployed a female-oriented pedagogy that increased female science students' positive attitude toward self-concept in science, enjoyment of science, and perception of science teacher. Female science students also developed less anxiety about science.

Furthermore, five of the selected interventions revolved around the use of female role models and mentoring strategies (5/25). In this regard, Stake's (2006) quasi-experimental research demonstrated that boys' stereotypes toward women in science could be changed by exposing them to female role models and mentors, as well as to positive information about girls' and women's science abilities. In addition, Stoeger et al. (2013) experimental study confirmed the advantages of 1-year female mentoring program in increasing girls' STEM activity, self-assessment of knowledge of STEM topics, self-assessment of knowledge about STEM-related university studies and jobs, confidence in one's own STEM abilities, self-assessment of STEM competences, and academic elective intentions.

Likewise, Denner's et al. (2012) quasi-experimental investigation observed that an after-school summer program with professionals serving as virtual mentors increased girls' computing career goals, expectations for success with computing, the value they placed on computing and computing-related jobs, and their perceived parental support. Strikingly, Good et al. (2003) experimental study significantly boosted the performance of girls, minority, and low-income students by addressing the psychologically threatening nature of the math assessments. Students were mentored by college students who encouraged them to view intelligence as malleable or to attribute academic difficulties to the novelty of the educational setting. Finally, Wilson's (2019) quasi-experimental research focused on analyzing the effectiveness of a STEM program on students' and teachers' efficacy and attitudes toward STEM. While girls' STEM confidence increased over time, no relationship between teachers' preparation and self-efficacy in STEM and students' STEM confidence was observed. Additionally, girls and students from ethnic groups felt as confident as boys and students from non-ethnic groups with their ability to learn with STEM resources and equipment.

Two of the remaining selected interventions were counseling-oriented (2/25). That is, experimental studies by Falco et al. (2010) and Falco and Summers (2019) observed improvements in girls' career decision self-efficacy and STEM self-efficacy as well as in students' motivation, value, enjoyment, and confidence in mathematics after the counseling sessions.

Finally, only two of the selected interventions focused on increasing parents' engagement (2/25). Heddy's (2014) experimental study demonstrated that combining a Teaching for Transformative Experience in Science (TTES) and a parent involvement (PI) intervention potentially ameliorated the reduction in girls' STEM motivation. In Hyde et al. (2017) experimental research, hypothetical responses of mothers to their children's usefulness of math and science classes increased adolescents' perception of math ability in seventh grade. Those responses also positively predicted adolescents' STEM interest in 10th grade.

In conclusion, the majority of the selected studies targeted personal factors through changes at the school level (*n* = 18), the delivery of STEM training (*n* = 9), modifications in pedagogy and curriculum of STEM content (*n* = 5), the promotion of single-sex and coeducational school context (*n* = 2), or the use of counseling sessions (*n* = 2). The rest of the intervention studies targeted changes in personal factors through a series of strategies at the environmental level (*n* = 7). Whereas, most of these used female role models (*n* = 5), the rest promoted parental engagement (*n* = 2).

## Discussion

This article provides a scoping review of interdisciplinary interventions designed to increase young people's interest in STEM and more particularly girls' interest in those STEM fields where women are highly underrepresented like engineering and computer science (Cheryan et al., 2013; UNESCO, 2018; Sáinz, 2020). The findings of the present study expand current knowledge on how to measure the impact and effectiveness of interventions designed to increase young people's interest in STEM. It responds to all research questions through an analysis of the characteristics of intervention studies aiming at raising young people's interest in STEM (RQ1) and of the methods used to measure the effectiveness of the interventions (RQ2). In addition, 25 exemplar intervention studies were selected to illustrate different approaches to addressing the topic, which can be a source of inspiration for the design and implementation of future intervention studies (RQ3).

In line with previous reviews and with research conducted in some of the studies included in our review, the present study confirms the need for designing interventions that lead to changes at family and school levels. In this regard, this review provides scholars, practitioners, policy-makers, and the general public with practical evidence of intervention studies that have fully or partially succeeded through the use of different strategies at the environmental and school levels in changing various personal aspects involved in shaping girls' and young people's interest in STEM.

However, and in comparison to previous reviews in this area, the present study provides a broader scope in the type of included publications. On the one hand, the 20-year time framework of this review is wider-ranging than earlier reviews. On the other hand, 25 dissertations were part of the initial review, but only five were included in our selection of exemplars. One of the strengths of the present review is a recognition of the theoretical foundations inspiring the methodological designs involved in the evaluation of the effectiveness of the interventions. Many of the identified characteristics of both publications and interventions deal with the methodology used to conduct the different studies, including the type of mixed method design, the typology of quantitative or qualitative research methods and techniques, as well as the kind of quantitative design used to collect, analyse, and interpret the collected data. All these features have not been comprehensively taken into consideration by earlier research on the effectiveness/impact of the evaluation of intervention studies. This is especially true in the case of the methodological design associated with interventions that have applied a mixed method approach.

### Measurement of the effectiveness of interventions to reduce the gender gap in STEM

A surprising amount of intervention studies did not have a stated theoretical foundation. This calls into question both the validity of designs and outcomes along with the extent to which the components of intervention evaluation have been fully comprehended (Liben and Coyle, 2014; Lazowski and Hulleman, 2016; Rosenzweig and Wigfield, 2016). In this review, motivation theories like expectancy-value, social learning, theories of intelligence, as well as constructivist theory shape most of the researchers' understanding and design of the studies addressing outcomes involved in shaping young people's interest in STEM pathways (Rosenzweig and Wigfield, 2016). Most of the studies included in this review use various constructs to measure issues associated with STEM achievement, STEM motivation, gender stereotypes about STEM careers, STEM identity, and emotional response toward STEM fields (Rosenzweig and Wigfield, 2016; van den Hurk et al., 2019).

These studies describe the different intervention practices and strategies used to target these constructs at the social-environmental (i.e., the use of female role models) and school levels (i.e., the inclusion of STEM training, changes in STEM pedagogy, the use of counseling sessions, or the influence of single-sex & co-educational contexts). Interestingly and in line with Rosenzweig and Wigfield (2016) findings, a great number of the studies informed about changes in various personal factors. However, most of them did not develop a proper theory of change explaining the psychological, social, environmental processes involved in this change.

Although a qualitative appraisal of interventions was not applicable due to the high number of publications covered, the identified interventions applied quantitative research methods. Random assignment was not possible for some of the interventions, due to the ethical constraints associated with educational fieldwork (i.e., Brock, 2017). That is, the random assignment of participants to experimental groups was not possible because the groups were already predefined previously to the study in classes or grade levels, and researchers could not reorganize them to meet the needs of the research (Mertler, 2016; Brown, 2019). Although several of the intervention studies applied a quasi-experimental design, a considerable number of them did not include a control group.

Interestingly, several studies combined qualitative and quantitative research methods. This reinforces the importance of using both methodological approaches when tackling complex phenomena, such as the underrepresentation of women in STEM. In contrast to previous systematic reviews (Rosenzweig and Wigfield, 2016; van den Hurk et al., 2019; Prieto-Rodriguez et al., 2020), studies using a mixed-method approach for measuring the effectiveness of interventions were included. Most of these studies used a convergent design (Creswell and Plano Clark, 2018) in order to gain complementary insights from the quantitative and qualitative findings regarding how, why, and under what conditions a particular intervention was successful or not in raising young people's interest in STEM.

Our scoping review highlights the need to design and include qualitative methods to evaluate the impact and effectiveness of interventions. This will enable a richer evaluation of the true influence on a targeted group. For instance, in Werner's (2017) study whereas quantitative data gauged the degree of change in six facets of attitude among female students, interviews were also used to gain deeper insights into student perspectives.

### Exemplary interventions that address the gender gap in STEM participation

A selection of 25 interventions of various types has been provided in this review, affording a useful reference point for the design of future research that can distinguish between short-term and long-term effects of interventions.

Since our research had a clear focus on studies evaluating intervention effectiveness, quantitative methods were used as an inclusion criterion for the exemplar studies as long as they allow researchers to accurately assess effectiveness, measuring changes in outcomes before and after the intervention (Mertler, 2016). Additionally, we recognize the value of qualitative methods, particularly when combined with quantitative methods in mixed methods designs. Especially when they aim at achieving additional evaluation objectives, such as determining the acceptability and feasibility of the intervention or determining how participants perceived the effectiveness of the intervention (Fetters and Molina-Azorin, 2020).

In line with van den Hurk et al. (2019) review, most of the identified intervention studies targeted various personal level factors linked to young people's interest in STEM, such as attitudes toward STEM learning, STEM self-efficacy, and self-perception of competence, or STEM achievement. Whereas, some of the reviewed studies employed strategies that modified environmental factors such as the increase of parental engagement or the use of female role models, other studies developed strategies that changed school level factors such as the inclusion of pedagogies in the teaching of STEM subjects, or the promotion of co-educational classrooms. This is another evidence of the complexity of the phenomenon associated with women's under-representation in several STEM pathways. In this regard and as shown in previous systematic reviews (Rosenzweig and Wigfield, 2016; Prieto-Rodriguez et al., 2020), most interventions used several constructs when raising girls' and young people's interest in STEM.

Incidentally, several of the selected interventions targeted personal factors, including self-competence beliefs identity formation sense of belonging, attribution of STEM-related success or failure, interest in STEM, and achievement, by modifying various aspects either at school (such as changes in STEM curriculum, the content of STEM training programmes, and teaching strategies) or at social-environment level (such as valuing women in science, or making visible female role models). Curiously, in some studies personal factors also tend moderate the effect that other factors of the intervention have on different indicators of STEM education. For instance, in Brock's study the efficacy of the intervention to increase math achievement was moderated by the students' sense of belonging. Similarly, in Todd and Zvoch (2019b) study, positive attitudes alone were not enough to increase girls' persistence in STEM. Self-efficacy and identities in science were also needed.

Interestingly, most of the selected interventions achieved their aims, whether it was closing the gender gap in STEM achievement or increasing interest in STEM particularly among female students. These interventions accomplished their goals by increasing the value placed on women in science improving female students' self-perception of STEM and encouraging them to pursue and persist with STEM, increasing students' self-efficacy in STEM, raising girls' interest in STEM by changing stereotypical images of computer science, retraining attribution toward physics, and increasing science affinities among female students.

Despite their qualities, several of the selected interventions demonstrated only partial effectiveness. For instance, Werner's (2017) intervention using female-oriented teaching strategies was insufficient to increase female science students' motivation. In a similar vein, Todd and Zvoch's (2019) intervention demonstrated that positive attitudes toward identity and self-efficacy among girls were not enough to increase persistence in STEM. This finding suggests that the impact of interventions addressing persistence in STEM should be measured longitudinally. Similarly, despite increasing interest in computer science among female students of color, the intervention by Scott et al. (2017) was unable to fully close the gender gap in interests and aspirations. This last finding confirms the importance of measuring long-term effects of the interventions implemented, especially in fields like computer science, where the participation of women is really scarce.

Moreover, a proper design of the interventions involves uunderstanding and anticipating the dynamics between early subjective STEM experiences and social and/or environmental challenges to STEM education (Schoon, 2001). In fact, in Schilling and Pinnell's study there were few opportunities for female participants to fully participate in engineering-related activities in the mixed gender group (i.e., they were given tasks associated with feminine roles, such as taking notes for the group), The inclusion of moderating factors also introduced complexity in assessing the real effect of the interventions. In Mayberry's (2015) study, after the changes made in the curriculum of science parental and educational background had no effect of on girls' interest, confidence, desire to learn about STEM and motivation to pursue STEM careers.

Many of the selected studies measured the effect of the intervention on more than a single construct. However, the intervention did not result in significant effects of the intervention on all the considered constructs. In Denner's et al. (2012) study, whereas the intervention with female virtual mentors had effect on some of the constructs under research (i.e., interest in computing jobs, confidence in computers, computer use, or perceived support from parents), girls' interest in problem-solving, endorsement of gender stereotypes, and perceived support from peers and teachers did not change. In Chiu's (2011) study the intervention focused on changing STEM pedagogy had no effects on students' attitudes toward science who participated in the experimental group.

### Limitations

This study has several limitations. First, despite the systematic and rigorous search strategy, we might have missed relevant intervention studies published in books or dissertations not indexed in the selected databases. Second, in accordance with the disciplinary background of the authors of this review, most of the journals included as part of the complementary search strategies were from the social, educational, and behavioral sciences, while journals from other STEM-related domains, such as engineering or from disciplines like human resource management, or economics were not used in the hand search strategy. Nonetheless, this does not imply that research from these fields was omitted from the review, as no discipline-specific database search terms or exclusion criteria were used. Third, consistently with the scoping review methodology (Pham et al., 2014), the balance between breadth and depth of analysis was a challenge in our review. Since our selection criteria were broader than in previous systematic reviews, we could not provide a detailed appraisal of each of the reviewed interventions individually because of the large number of included publications. Fourth, the broad scope of the review also limited the depth of analysis in the appraisal of the interventions' effectiveness. Fourth, commensurate with the recommendation of privileging the comprehensive coverage of the literature over critical appraisal in scoping reviews, the methodological rigor of the included studies was not appraised.

### Implications and recommendations

The present study highlights the important role that theory plays in the design and evaluation of the interventions to reduce the various gender gaps in STEM. However, and considering Yeager and Walton (2011) conclusions, the success of several of the interventions highly relies on societal and educational contexts. The theoretical and practical implications of this review on the effectiveness of interventions in the STEM field are numerous and can be a source of inspiration, both for the design of future interventions aimed at promoting the interest of girls in STEM fields and for their evaluation.

The high number of the identified interventions that focus on raising interest in STEM fields among girls and boys not only denotes a lack of STEM talent, but also the necessity of targeting both genders and incorporating a gender dimension in raising and retaining young people's interest in STEM. This issue not only relates to the underrepresentation of women in STEM, but it also involves aspects relating to the role that women play in STEM fields in particular and in society in general.

Similarly, since longitudinal evaluations do not necessarily demonstrate that interventions promoted long-term engagement in STEM (Prieto-Rodriguez et al., 2020), it is essential that this type of research measures a long-term engagement and not just simply a follow-up. Interestingly, in this review the benefits of combining qualitative and quantitative research methods in the design and implementation of interventions are highlighted. This implies future avenues for interdisciplinary collaboration in the design and implementation of interventions to raise women's interest in STEM.

In addition, given that most of the published interventions have been systematically conducted in the US (Liben and Coyle, 2014; Rosenzweig and Wigfield, 2016), more publications should be promoted tackling interventions implemented in different international countries with less international visibility of the initiatives and efforts implemented to fight against women's underrepresentation in STEM. These could also provide further cultural insights into how to improve the effectiveness of interventions addressing gender gaps in STEM pathways.

Interestingly, the combination of afterschool and within school activities could be an extraordinary way of increasing the likelihood of the interventions to improve women's attraction and retention in the STEM pathway over time. Many interventions took place in the classroom, through games, counseling sessions, and hands-on activities, and deployed various activities consistent with the STEM curriculum. Others were organized as extracurricular activities, such as summer camps and various afterschool activities, and incorporated diverse leisure activities, a highly important aspect in encouraging young people's vocational interests. These indicate the wide range of possibilities that both extracurricular and classroom activities have for the formulation of significant, innovative, and grounded interventions that can reach long-term goals.

From the results of the present review, we can assume that the inclusion of a gender perspective is a key element when analyzing the potential effect of gender issues in the way the interventions are designed and evaluated. Interventions must target both genders, as boys should also be exposed to interventions with female role models and learn more about contributions by women within STEM (Werner, 2017; González-Pérez et al., 2020). It is crucial that both girls and boys learn how existing gender roles about academic competences influence career choices. As noted by Good et al. (2003) and Stake (2006), the powerful influence of stereotype threat means that male bias may significantly limit the science performance of girls and women as well as their willingness to choose and persevere in STEM fields. Therefore, it is important that we understand the basis for negative attitudes toward women in STEM. As Harackiewicz and Priniski (2018) noted in their systematic review of interventions in higher education, if the design of interventions is not undertaken correctly, students of both genders may become disengaged and abandon STEM pathways.

Most of the interventions focused on increasing STEM interest, mainly in the fields of science, mathematics, and computer science, but a few of them attempted to increase interest in STEAM fields. This suggests that interventions combining the intersection between STEM competences and competences beyond STEM are needed. As Schilling and Pinnell (2019) study noted, some of the intervention studies failed to reflect the diversity of participants, as information about ethnicity and/or social origin was not gathered in many cases. Therefore, an intersectional analysis has not been conducted in most the intervention studies. For this reason, future design should incorporate various aspects relating to intersectionality and how it can help in properly addressing various gender gaps in STEM.

The duration of interventions might inform about their sustainability and impact of the intervention. Long-term exposure to STEM is needed to properly address the underrepresentation of women in STEM pathways. Ideally, more follow-up research is required in order to determine the long-term effect of interventions and the extent to which they are effective in increasing the number of high school and university STEM enrolments, as well as retain women and students with different characteristics in STEM pathways.

Future research should provide more rigorous theoretical foundations in the design and development of intervention studies. This would have an impact on the outcomes to be measured and the evaluation process. Follow-up studies with Randomized Controlled Trials (RCTs) and other research designs are required to fully comprehend the development of student career choices and preferences across STEM. The inclusion of variables beyond gender is also necessary since they provide further insights into the participation of women and other minorities in STEM pathways.

## Data availability statement

The original contributions presented in the study are included in the article/Supplementary material, further inquiries can be directed to the corresponding author.

## Author contributions

MS and SF: review conceptualization, design, search strategy, and writing—original draft. MS, SF, MR, and B-SL: screening, study eligibility, data extraction and analysis, review, and editing. All authors contributed to the article and approved the submitted version.

## Funding

The present study belongs to a research project led by the MS funded by the Spanish Ministry of Industry, Economy, and Competitiveness. Spanish State Research Agency (AEI) and European Regional Development Fund (ERDF) (Grant No. FEM2017-84589-R).

## Conflict of interest

The authors declare that the research was conducted in the absence of any commercial or financial relationships that could be construed as a potential conflict of interest.

## Publisher's note

All claims expressed in this article are solely those of the authors and do not necessarily represent those of their affiliated organizations, or those of the publisher, the editors and the reviewers. Any product that may be evaluated in this article, or claim that may be made by its manufacturer, is not guaranteed or endorsed by the publisher.
