# Supplementary material for: Interventions to increase young people's interest in STEM. A scoping review
Source: Front Psychol. 2022 Oct 11;13:954996. doi: 10.3389/fpsyg.2022.954996 (PMC9593045; doi:10.3389/fpsyg.2022.954996)
Supplement: Supplementary file 1 [file Table_1.pdf]

Supplementary Material

Table 1_v1. Best interventions to raise girls’ interest in STEM

Intervention	Main Purpose	Theory	Quantitative Design	Gender	Target	Strategy	Context	Main Findings
Brock, 2017	-To assess the gender gap in mathematics achievement, self-efficacy, and sense of belonging and determine if an all-girls supplementary mathematics class could help to close it	Various	Quasi-Experimental (Within a mixed methods design)	Boys/ Girls	Only Students	STEM Training	Ordinary Classroom Workshops	-The intervention helped to close the gender gap.<br>-High ability girls gained more on the standardized mathematics test than high-ability boys.
Cantley et al., 2017	-To explore how the collaborative cognitive activation teaching strategies in mathematics improve girls’ attitudes to mathematics.	Various	Quasi-experimental	Boys/ Girls	Only Students	Curriculum and Pedagogy Improvements	Ordinary Classroom Hands-on Activities	-Small improvements in girls’ interest in and enjoyment of mathematics, but no significant effect for boys.<br>-No significant difference between girls’ and boys’ pre-intervention enjoyment scores.
Chiu, 2011	-To increase girls’ positive attitudes towards learning science by valuing women in sciences	Holland’s Career Development	Experimental	Boys/ Girls	Only Students	Curriculum and Pedagogy Improvements	Ordinary Classroom Hands-on Activities	-The gender gap favored boys in the pretest for both the experimental and control groups with regards the value of learning science, while it favored girls in the posttest only for the experimental group
Denner, 2007	-To examine the effects of the Girls Creating Games program to increase the capacity of participants to pursue and persist with computer	Expectancy Value	Quasi-Experimental (Within a mixed methods design)	Only Girls	Only Students	STEM Training	Extracurricular Afterschool Activities	-Self-perception of computer skill increased in the intervention group, and was higher for the treatment.<br>-The treatment group had an increase in their perception of knowledge about computers.<br>-The treatment group decreased stereotypes regarding boys do better using computers, whereas the comparison group increased theirs.
Denner et al., 2012	-To increase middle school girls’ interest to pursue courses and careers in computing and to increase their belief that they could be successful at computing activities.	Expectancy Value	Quasi-Experimental (Within a mixed methods design)	Only Girls	Only Students	Role Model/ Mentoring	Extracurricular Afterschool Activities	-More girls were interested in computing jobs<br>-A significant increase in computing skills, confidence with computers and frequency and range of computer use.<br>-Increased perceived parental support to pursue science and/or computing careers.

								-Increase in some stereotypical views of computer scientists. However, the stereotypical images of computer scientists decreased.
Drobnis, 2010	-To examine the impact of an all girls' classroom environment in a high school introductory computer science class on the students' attitudes towards computer science and their thoughts on future involvement with computer science.	Feminist Theories	Quasi-Experimental (Within a mixed methods design)	Boys/ Girls	Only Students	Single-Sex & Co-Educational Context	Extracurricular Summer Camps	-Male students and girls from the all girls' class had a greater increase in efficacy than girls from the mixed gender classes. Male students had the greatest increase<br>-All students had an increase in taking more computer science in high school.<br>-The girls from the mixed gender sections had no gain in using CS in a future career, while the girls from the all girls' class and the boys had considerable gains.
Falco & Summers, 2019	-To evaluate whether a career group intervention that incorporates the four sources of self-efficacy and addresses perceived career barriers is effective at improving the career decision self-efficacy and STEM self-efficacy for adolescent girls	Social Learning	Experimental	Only Girls	Only Students	Counselling -Oriented	Ordinary Classroom Counselling Sessions	-No differences between the experimental and control groups at Time 1, but significant differences between groups after the intervention for career decision self-efficacy and STEM self-efficacy.<br>-Students in the experimental group improved on both career decision self-efficacy and STEM self-efficacy 3 months after the intervention
Falco et al., 2010	-To increase students' interest and engagement in mathematics.<br>-R.Q.1. will the school-counselor-led curricular intervention have an effect on students' self-efficacy, mathematics performance, and attitudes towards mathematics learning?<br>-R.Q.2. will the intervention have a different effect for girls than for boys?	Various	Experimental	Boys/ Girls	Students & Teachers	Counselling -Oriented	Ordinary Classroom Counselling Sessions	-Intervention improved students' attitudes toward mathematics learning (particularly for girls) and performance.<br>-Students enrolled in classes with the most experienced math teacher had higher self-efficacy.<br>-Girls in the experimental group had higher growth
Good et al., 2003	-To test methods of helping female, minority, and low-income adolescents overcome the anxiety inducing effects of stereotype threat and, consequently, improve their standardized test scores	Various	Experimental	Boys/ Girls	Only Students	Role Model/ Mentoring	Ordinary Classroom Hands-on Activities	-Stereotyped students -females in math, and ability-stigmatized students in reading -increased their standardized test scores.<br>-Boys outperformed girls on the math test if they had been mentored in the harmful consequences of drug use.<br>-When participants learned about the expandability of intelligence the gender gap in math performance disappeared

Hall-Lay, 2018	-To compare STEM-related self-efficacy scores among male and female students who participated in OST robotics programs or other OST STEM-related programs.	Social Learning	Quasi-Experimental	Boys/ Girls	Students	STEM Training	Extracurricular Afterschool activities	-Students in the OST Robotics programs had higher STEM-related self-efficacy. The relationship between STEM-related self-efficacy scores and type of OST programming was not moderated by gender
Heddy, 2014	-To explore whether the utility of Teaching for Transformative Experience in Science (TTES) model could be enhanced through a parent involvement intervention for developing middle and high school girls' interest in STEM.	Expectancy Value	Experimental (Within a mixed methods design)	Only Girls	Students & Parents	Parental Engagement	Ordinary Classroom Hands-on activities	-TTES with the addition of a parent intervention facilitated more out-of-school engagement and parent involvement than a comparison.<br>-The addition of a parent may increase the effectiveness of TTES and maintain girl's interest in science.
Hyde et al., 2017	-To investigate mothers' communication with their adolescent regarding usefulness of science and math classes in high school.	Various	Experimental	Boys/ Girls	Students & Mothers	Parental Engagement	Extracurricular Afterschool activities	-Adolescents whose parents received the intervention reported more UV in 10th grade than those whose parents were in the control group.<br>-Adolescents whose parents received the intervention took more math and science courses, compared with controls.
Isiksal & Askar, 2005	-To investigate the effect of dynamic geometry software on the mathematics achievement and mathematics self-efficacy of 7th-grade students.<br>-Also, it further examines the gender differences with respect to computer self-efficacy, mathematics self-efficacy and mathematics achievement.	Social Learning	Experimental	Boys/ Girls	Only Students	STEM Training	Ordinary Classroom Hands-on activities	-The Autograph group gain scores were higher in mathematics self-efficacy<br>-Boys scored significantly higher than girls in computer self-efficacy.<br>-No difference was found on gain scores of girls and boys with respect to mathematics self-efficacy and mathematics achievement.
Mayberry, 2015	To explore if a three-week Girl Scout curriculum would improve the interest and confidence of urban female students in STEM careers.	Various	Quasi-experimental (Within a mixed methods design)	Only Girls	Various (students, teacher, parents, mentors)	Curriculum and Pedagogy Improvements	Extracurricular Workshops, Presentations	-The workshop did increase the interest of the Girl Scouts in STEM<br>-There was an increase in the Girl Scout's self-confidence in science.<br>-Parents and troop leaders responded that the girls learned new words related to STEM.<br>-The Girl Scouts felt that girls and boys would be equally successful in completing STEM jobs.

McHugh et al., 2018	-To explore whether students' understanding of science content improved as a result of a mathematics-infused science curriculum, and if these gains were experienced equitably in terms of gender and SES.<br>-If students' views on the relationship between mathematics and science changed as a result of the intervention.	Constructivist and Learning-Related	Quasi-experimental	Boys /Girls	Only Students	Curriculum and Pedagogy Improvements	Ordinary Classroom Workshops	-Scores on the Science Knowledge posttest were higher for students in the infusion group<br>-Females outperformed males in science but no differences in achievement between students from high and low needs schools.<br>-Students in the treatment group increased their confidence in performing science tasks and their usefulness of mathematics in learning science
Paslov, 2006	-To improve girls' attitudes towards and achievement in mathematics.	Expectancy Value	Quasi-Experimental (Within a mixed methods design)	Boys/ Girls	Students and Teachers	STEM Training	Ordinary Classroom Hands-on Classroom Activities	-Both female and male experimental groups had improvements<br>-When males and females were not disaggregated in the experimental group and the control group, no difference was noted in overall change in attitudes<br>-Females in the experimental group outperformed the control group in 3 of the 7 attitude scales.<br>-Males in the experimental group showed greater gains or smaller reductions than males in the control group<br>-The experimental group males were not impacted by curriculum in any of the attitude. However, the experimental group of females was
Schilling & Pinnell, 2019	-To evaluate the effects of single-sex female and co-education engineering camps on female participants' interest and self-efficacy in engineering.	Social Learning	Experimental	Boys/ Girls	Only Students	Single-Sex & Co-Educational Context	Extracurricular Competitions	-An increase between the pre and post-camp self-efficacy responses<br>-For the female participants from the co-ed engineering camp group questions about their confidence in their math and science skills and likelihood of pursuing engineering showed significance
Scott et al., 2017	-To examine a double-bind affecting the outcomes of women of color in computing, with the research questions:<br>-Do gender differences exist in computer science interest and aspirations?<br>-Do existing gender differences persist in participation in CS courses in high school and the pursuit and completion of computer science degrees?	Feminist Theories	Quasi-Experimental (Within a mixed methods design)	Boys/ Girls	Only Students	STEM Training	Extracurricular Summer Camps	-Females were less likely to be engaged in CS.<br>-Although girls represent 53% of participants, just 23% listed computer science as their "favorite," compared to 39% of male participants.<br>-50% of girls listed computer science as their favorite class were in their second year of the program<br>-Girls of color started out with less interest in computer science, but this interest grew over time.<br>-The intervention was unable to close the gender gap in interests and aspirations in CS.

Stake, 2006	-To explore the basis for boys' negative attitudes toward women in science among high school students with aptitude and motivation for a science career.	Stereotype Threat	Quasi-experimental	Boys/ Girls	Only Students	Role Model/ Mentoring	Extracurricular University Camps	-Neither boys nor girls gave higher ratings on any Attitude-related item following the program.<br>-At the beginning, more negative attitudes toward women in science were linked to lower science self-confidence among boys, but not among girls.<br>-Changes in self-confidence over the course of the program were related to changes in attitudes toward women in science among boys but not among girls.
Stoeger et al., 2013	-To increase the participation rates of girls in STEM.<br>-To evaluate the effectiveness of the program with regards to the goals of increasing the frequency of girls' activities in STEM.	Social Learning	Experimental	Only Girls	Only Students	Role Model/ Mentoring	Extracurricular Out of school activities	-Self-assessment of knowledge about university studies and jobs in STEM increased across time for treatment group.<br>-Values for the treatment group remained the same across the three measuring points.<br>-Self-assessment of STEM competencies decreased significantly for the control group, but it remained roughly the same at each of the three measuring points for the treatment group.<br>-Academic intentions increased in the treatment group over time, but remained stable in the control group.
Todd & Zvoch, 2019a	-To determine if participation in an informal science outreach program built around the theories of identity formation and self-efficacy in middle school girls' science affinities.	Various	Experimental	Only Girls	Only Students	STEM Training	Extracurricular Summer Camps	-Intervention participants scored higher than their control group peers<br>-Of the affinity outcomes, science efficacy and science attitude were most consequential.
Todd & Zvoch 2019b	-To investigate whether participation in an informal summer science program was associated with a positive short-term change in middle school girls' science affinities<br>-To examine girls'perceptions regarding science norms and practice.	Various Theories	Quasi-Experimental (Within a mixed methods design)	Only Girls	Only Students	STEM Training	Extracurricular Summer Camps	-The variability in science affinity outcomes was associated with girls' background characteristics and experiences.<br>-Identity and self-efficacy played a larger role in distinguishing the groups than attitudes toward science.<br>-For girls, positive attitudes are not enough to increase persistence in STEM, thus highlights the importance of self-efficacy and identities in science.

Werner, 2017	- To analyze if female-oriented teaching strategies facilitate a change in attitude towards the discipline of science of middle school female students.<br>-Which strategies do help to improve their attitude toward science?	Various	Quasi-Experimental (Within a mixed methods design)	Only Girls	Only Students	Curriculum and Pedagogy Improvements	Ordinary Classroom Hands-on Activity	-The female had high regard for the science teacher and the value of science. When a more female-oriented pedagogy was implemented, the female science students developed more positive attitude in self-concept in science, anxiety about science, enjoyment of science, and perception of science teacher.<br>-Implementation of female-oriented teaching strategies did not sufficiently enhance the motivation of the female science students.<br>-The participants responded most favorably to being taught by guided inquiry, the introduction of female science role models, working collaboratively in single-sex groups, and learning about the relevance of science.
Wilson, 2019	-To prove the effectiveness of a STEM program by exploring the relationship between teacher efficacy in STEM and students' confidence in and attitudes toward STEM.<br>-To analyze whether a difference existed in teachers and students surveyed before and after the program, and whether a difference existed in terms of race and gender regarding levels of STEM confidence.	Constructivist and Learning-Related	Quasi-Experimental (Within a mixed methods design)	Girls/ Boys	Students & Teachers	Role Model/ Mentoring	Ordinary Classroom Hands-on Activity	-No significant relationship between teachers' preparation and self-efficacy in STEM and students' confidence in STEM.<br>-An increase in teachers and students' STEM efficacy and confidence<br>-No differences regarding gender or race in STEM students' confidence and attitudes<br>-Females' STEM confidence and attitudes increased
Ziegler, & Heller, 2000	To test the effects of an Attribution Retraining program on gifted female students' motivation and performance in Physics.	Attribution	Experimental	Only Girls	Only Students	STEM Training	Ordinary Classroom Hands-on Activity	-Treatment group had higher attributions of success/ failure to effort, and lower to task difficulty, for success and failure<br>-Lower perceived helplessness; Higher perceived control over successes and failures; Higher interest in physics; Higher desire for career in physics; Higher desire to pursue a concentration in physics.<br>-Treatment group had higher physics test scores
